# A Brief History of Otology from Its Infancy to the Present Time

**Published:** 1896-04

**Authors:** T. E. Mitchell

**Affiliations:** Columbus, Ga.


ATLANTA

### Medical and Surgical Journal.

VOL. XIII. APRIL, 1896. No. 2.

LUTHER B. GRANDY, M.D.,
MANAGING EDITOR.

M. B. HUTCHINS, M.D.,

BUSINESS MANAGER.

COLLABORATORS:
H. V. M. MILLER, M.D., LL.D., A. W. CALHOUN, M.D., LL.D., VIRGIL O. HARDON, M.D., FLOYD W. McRAE, M.D., AND DUNBAR ROY, M.D.

### ORIGINAL COMMUNICATIONS.

### A BRIEF HISTORY OF OTOLOGY FROM ITS INFANCY TO THE PRESENT TIME.

BY T. E. MITCHELL, M.D.,
COLUMBUS, GA.

Perhaps no branch of medical science has been longer in receiving the consideration its importance merits than has that of otology, or, the knowledge of the anatomy, physiology, and pathology of the ear. While this is true, it is likewise a fact that the earliest physicians prescribed for diseases of the ear, notwithstanding their remedies were for the most part empirical and irrational.

Hippocrates, who died 357 B.C. and who was seventeenth in descent from Æsculapius himself, had an indefinite knowledge of the membrana tympani, and considered all internal inflammations of the ear as inflammations of the head. He recognized pain in the ear accompanied by high fever as a very dangerous malady, which usually terminated in the death of the patient in about ten days unless pus escaped from the ear or blood from the nose. In constitutional troubles Hippocrates regarded sweet ear-wax as an unfavorable symptom. Among the many strange preparations which he instilled into the ear for the relief of pain, we find a very efficient one in warm water, though it is probable that he knew nothing of the rationale of its anodyne properties. Alcmeon, who lived about 530 B.C., was probably the first man to dissect the human cadaver, and is said to have known something of the ear and Eustachian tube. Aristotle (385–322 B.C.), a pupil of Plato, was not only a philosopher, but an anatomist as well. Much of his more mature life was spent with a tenaculum and scalpel in making anatomical investigations on animals. He had an indefinite idea of the myringa and Eustachian tube. Popular remedies at this time were burned opium and castoreum for earache, and bitter almond oil for living creatures. Asclepiades, a contemporary and friend of Cicero, advised instillation of oil in which cockroaches had been cooked, and this to be followed by woman's milk. Celsus, a Latin physician, who practiced during the reign of Augustus, prescribed indiscriminately a remedy composed of cinnamon, cassia, castoreum, white pepper, ammonia, myrrh, and saffron, rubbed up and diluted with vinegar, for all ear diseases. Celsus was probably the first to suggest the removal of foreign bodies from the ear by vigorous injections of water. In obstinate cases which did not yield to the method above named he recommended an alternative no less ingenious than unpleasant to the patient, who was to be laid on a table with the affected member down, whereupon the physician should strike the table with a hammer, hoping to dislodge the body by concussion. Archigenes, who lived about this time, recommended the local application of hot water, purgative enemas, and venesection for aural pains. To remove a foreign body from the ear of a child, he suggested that it be held up by the feet and given a vigorous shake, while adults should be treated in a similar manner to that recommended by Celsus. He was probably the first to suggest the use of speaking tubes for the deaf. Rufus, who practiced in the historic city of Ephesus in the first century, gave to the different parts of the auricle the names of helix, lobe, tragus, and anti-tragus, which names have been retained.

Galen (A.D. 130–201), the most celebrated physician of his time, was an elaborate writer, but added little or nothing to what was already known of the anatomy of the ear. Galen made an extensive classification of the diseases of the ear, and countless remedies were among his therapeutic resources. Among these were wolf's milk, the fat of geese and hens, or, when the pain was severe, a mixture of opium, musk, and the white of an egg. From the time of Galen (A.D. 200), very little or nothing was added to the indefinite and superficial knowledge of the anatomy of the ear till the sixteenth century; but much was written of aural therapeutics, most of which was based on ignorance, superstition, and empiricism. A marked exception to the popular remedies of the time was found in the teachings of one Aurelianus, who lived after Galen and who suggested the use of leeches, cups, sinapisms, etc., for pain in the ear. Some of the strange and unreasonable things that were poured into the ear for various troubles were, frog's fat for pain, the renal secretion of pigs, men, or children, and the blood of young chickens, for an ulcer. A great deal was written of living larvæ in the ear, the frequency of their occurrence being probably due to neglected or improperly treated suppuration of the middle ear. For this trouble the saliva of a hungry man was a favorite prescription. For deafness, a popular prescription was a lion's brain mixed with oil, and one disciple of Æsculapius found an efficient remedy for the earache of children in woman's milk, provided, however, that the milk be that of a woman nursing a female infant, in case the patient be a boy, and *vice versa* if it be a girl. For 1,300 years immediately subsequent to Galen's time no progress was made in otology, and those who practiced during this time groped in a darkness of ignorance, superstition, and empiricism only to leave this branch of medical science in as undeveloped a condition as they found it. During the sixteenth century, however, great strides were made in anatomical researches of the ear, and a more perfected knowledge of its parts was handed down to subsequent investigators.

Vesalius (1514–1561), a learned anatomist, described the vestibule, the semi-circular canals, and the small tube connecting the tympanic cavity with the pharyngeal space.

Eustachius (1510–1574), an Italian anatomist, discovered the tensor tympani and stapedius muscles, as well as to describe more clearly the tube which bears his name, thus perpetuating his memory in anatomical nomenclature.

Fallopius (1523–1562), likewise an Italian anatomist, explained the communication of the tympanic cavity with the mastoid cells, described the fenestræ ovalis and rotunda, as well as the canal (aquæductus Fallopii) which bears his name and through which the facial nerve passes. It was about this time that the ossicula auditus was discovered.

Among other and less famous anatomists who contributed to our knowledge of aural anatomy about this time were Ingrassias, Colombo, Casserius, Stenon, and Du Verney. Valsalva spent sixteen years of his life in the study of otology, and in this time dissected more than one thousand heads. As a result the eighteenth century was a great era in aural anatomy. Valsalva was soon followed by Scarpa (1747–1832), who took up the chain of anatomical investigation where his predecessor had left it; and so exact were his descriptions of the structure of the inner ear that his contemporaries thought little else was to be done. Bivinus, Casselbohm, Zinn, Brendel, Monro, and Cotugno were also contemporary anatomists of this time, and helped to complete our knowledge of the ear's mechanism. Cotugno was the first to show that the labyrinthin fluid was essential to sound perception.

Morgagni (1682–1771), the founder of pathological anatomy, did much for otology by opening up a field for investigation without a knowledge of which therapeutics must necessarily be irrational and empirical. We are now well into the nineteenth century with our anatomical investigations of the ear, and find a host of workers eliminating the false and assimilating and classifying the accumulated facts of centuries, as well as adding others as the result of their own labors. Prominent among the anatomists of the nineteenth century who have apparently perfected our knowledge of the anatomy and physiology of the ear are, Shrapnell, Wharton Jones, Huschke, Breschet, Bock, Arnold, Jacobson, Muller, Toynbee, Politzer, Corti, and Hyrtl, the latter of whom called attention to the frequent porosity of the thin bony partition between the tympanic cavity and the brain, thus explaining how an otitis media may develop a meningitis. Pathological anatomy of the mastoid cells and its relation to diseases of the tympanum and brain are receiving quite a good deal of attention at this time. We have to regret that aural therapeutics never kept abreast with the progress of anatomical researches, and otological medicine, until within the last few decades, was in a great measure illogical and irrational. Fallopius taught that a discharge of pus from a child's ear was an effort of nature to remove obnoxious matter from the brain, and consequently should not be interfered with. This idea, false and irrational as it is, was studiously observed by some of his successors until comparatively recent times. Arcularius, a contemporary of Fallopius, had a rather unusual way of removing a foreign body from the ear. The head of a lizard is to be severed from its body and placed in the ear, and removed at the expiration of three hours, when the foreign body would be found in its mouth. One Capivacci, in the sixteenth century, devised a method of making a differential diagnosis between central and peripheric diseases of the ear by means of a vibrating iron rod held between the teeth. A modification of this test is found in the tuning-fork of to-day. Capivacci taught that deafness due to disease of the auditory nerve was incurable, which, for the most part, we find to be true at this time.

Sassonia, of the seventeenth century, is responsible for the deep-rooted delusion that complete deafness invariably follows a rupture of the membrana tympani. Thomas Willis, in the latter part of this century, attempted to refute this teaching by experiments on animals. Willis likewise made the first recorded observations of that condition of things in which certain persons hear better in a noise. This phenomenon, which is usually observed in persons suffering with an otitis media catarrhalis chronica, is called, after its describer, *paracusis Willisiana*. Among the instances cited by Willis is that of a wife who could not hear her husband except in a great noise. Consequently a servant was kept in the house whose duty it was to beat violently a drum during a conversation between the heads of the household.

We have now come to the latter part of the seventeenth century, and find that, although much of the false has been eliminated and otology for the most part is on a higher plane than it was in earlier and darker days, still many authors continue to teach of strange and unreasonable decoctions and ear-drops. Among the therapeutic resources of one practitioner of this time, the renal secretion of a female donkey, a male hare, and a wolf or white goat was mixed, warmed, and used in the ear.

Passing on to the eighteenth century, we find Valsalva's contributions to otological therapeutics second only to his anatomical investigations. He taught, among other things, that the hearing is merely impaired when the tympanic membrane is ruptured, and that a membrana tympani is in some cases restored after a solution of its continuity. He was the author of the Valsalvian method of forcing air through the Eustachian tube, and recommended it as a means of removing pus from the middle ear. He also proved that impaired hearing is often due to closure of the Eustachian tube. In 1724 a French layman, Guyot, washed out the mouth of the Eustachian tube by means of a complicated contrivance introduced through the mouth, and thus improved his blunted hearing. Encouraged by Guyot's experience, Cleland, an English surgeon, in 1741, devised a means for injecting fluid through the nose into the mouth of the Eustachian tube, and in a short while the Eustachian catheter formed an essential part of every otological surgeon's armamentarium. But this valuable therapeutic agent soon fell into unmerited disrepute owing to the fact that all deaf ears were catheterized indiscriminately, when in many instances an unfavorable prognosis should have been given at the beginning. Surgical interference in mastoid troubles was about this time brought prominently beofre the profession, mainly by the observations of Petit; but injudicious discrimination in the selection of cases for operation caused *trephining the mastoid* to become unpopular and remain so to within comparatvely recent times. In the early part of the nineteenth century, an English physician (Saunders) wisely advised paracentesis of the membrana tympani in acute suppurative inflammation of the middle ear rather than leave it "to a casual ulceration."

It was at this time that Sir Astley Cooper introduced the operation of perforating the tympanic membrane for deafness due to chronic non-suppurative inflammations of the middle ear.

Dr. Yearsley, of London, in a treatise published in 1863 traced the origin of most cases of impaired hearing to a pathological condition of the mucous membrane of the nasopharynx. Dr. Yearsley was really the first to use an artificial membrana tympani, which was afterwards improved upon by Toynbee. William Wilde's treatise on the ear (1853) did much towards placing aural surgery on a rational basis. Recognizing that most diseases of the ear are inflammatory in nature, he inaugurated a system of antiphlogistic treatment, which, with some modification, is practiced to-day.

Toynbee, by his physiological investigations having demonstrated that the mouth of the Eustachian tube is opened by the act of swallowing, and that the naso-pharyngeal opening is closed, it was left for Adam Politzer, of Vienna, in 1862, to immortalize himself by promulgating his method of inflating the middle ear with air. This apparently simple procedure has probably restored the hearing of more ears than the majority of all other means combined; indeed, in properly selected cases it rarely fails to give satisfactory results. Prominent among the authors of the present century, not mentioned above, who have contributed materially to our knowledge of otology, are, Burnett, Buck, Gruber, Pomeroy, Dench, and Roosa; and so thorough have been their investigations that this branch of medicine offers an inviting field for study, and bids fair soon to be on a plane with ophthalmology. Some idea of the magnitude of this study may be had from the statistical reports of the New York and Brooklyn hospitals, which show that nearly one thousand aural patients are examined annually in these institutions.

Authorities consulted: Encyclopedias, journals, and various treatises on the ear, particularly Roosa.

9 *East* 11*th St.*

---

"WHAT are you doing?" Dr. Holmes once asked a student in the dissecting-room. "Ligating arteries, sir." "Why not say tie?" rejoined the doctor. "I find that country practitioners ligate the arteries and that surgeons tie them." The best of this anecdote is that the unappreciative student spread it as a joke against Dr. Holmes.—*Ex.*

